# Use of Testing for West Nile Virus and Other Arboviruses

**DOI:** 10.3201/eid2209.152050

**Published:** 2016-09

**Authors:** Jakapat Vanichanan, Lucrecia Salazar, Susan H. Wootton, Elizabeth Aguilera, Melissa N. Garcia, Kristy O. Murray, Rodrigo Hasbun

**Affiliations:** University of Texas Health Science Center at Houston, Houston, Texas, USA (J. Vanichanan, L. Salazar, S.H. Wootton, E. Aguilera, R. Hasbun);; Baylor College of Medicine, Houston (M.N. Garcia, K.O. Murray)

**Keywords:** meningitis, encephalitis, community-acquired CNS infections, West Nile virus testing, arboviral disease, viruses, vector-borne infections, arboviruses, WNV

## Abstract

In the United States, the most commonly diagnosed arboviral disease is West Nile virus (WNV) infection. Diagnosis is made by detecting WNV IgG or viral genomic sequences in serum or cerebrospinal fluid. To determine frequency of this testing in WNV-endemic areas, we examined the proportion of tests ordered for patients with meningitis and encephalitis at 9 hospitals in Houston, Texas, USA. We identified 751 patients (567 adults, 184 children), among whom 390 (52%) experienced illness onset during WNV season (June–October). WNV testing was ordered for 281 (37%) of the 751; results indicated acute infection for 32 (11%). Characteristics associated with WNV testing were acute focal neurologic deficits; older age; magnetic resonance imaging; empirically prescribed antiviral therapy; worse clinical outcomes: and concomitant testing for mycobacterial, fungal, or other viral infections. Testing for WNV is underutilized, and testing of patients with more severe disease raises the possibility of diagnostic bias in epidemiologic studies.

## Abstract 2

For patients with meningitis and encephalitis, testing for these viruses is underutilized.

Arboviruses (arthropodborne viruses) are viruses that can infect humans via arthropod vectors, including mosquitoes, ticks, and sand flies. In the United States, the most common arboviral disease is infection with West Nile virus (WNV), which is transmitted largely by mosquitoes of the genus *Culex*. Since the first detection of WNV in the United States in 1999, several outbreaks of WNV infection have occurred in cyclic patterns (*1*,*2*). Texas is considered to be an area where transmission of WNV is endemic and occasionally epidemic; to date, >4,000 clinical cases in Texas have been reported (*3*,*4*).

Most patients with WNV infection are asymptomatic, but uncomplicated West Nile fever develops in ≈20% (*5*). In contrast, West Nile neuroinvasive diseases (WNND) occurs in <1% of infected persons; however, a substantial proportion of illness and death occurs among these patients, who experience clinical manifestations such as long-term neuropsychiatric sequelae and chronic renal insufficiency (*6*–*11*). WNND is characterized by encephalitis and meningitis and sometimes (rarely) acute flaccid paralysis (*7*,*8*).

Acute WNV infection is diagnosed by detection of WNV-specific IgM in serum, cerebrospinal fluid (CSF), or both (*1*). The validation of commercial tests for serum WNV IgM demonstrated sensitivity of 86%–96% and specificity of up to 100% (*12*,*13*). Despite the availability of sensitive testing for WNV, serologic testing is often underutilized, probably because of lack of physician awareness. In a blood donor screening study, <50% of patients with symptomatic WNV infection sought medical care, and only 5% of them received a diagnosis of WNV infection (*5*). Moreover, in a small study of 60 patients with meningitis and encephalitis, only 40% were serologically tested for WNV (*14*). Underutilization of testing contributes to multiple biases (e.g., selection, misclassification) within epidemiologic studies, which are often used to drive public health policy and resources for mosquito control. Accurate data about patterns of WNV utilization in routine clinical practice is needed for enhancement and tailoring of future public health interventions.

## Methods

### Study Population and Case Definition

We performed a retrospective multicenter descriptive study of meningitis and encephalitis patients >2 months of age at any of 9 hospitals associated with Memorial Hermann Hospital in the greater metropolitan area of Houston, Texas, USA, from January 1, 2005, through December 31, 2010. The study was approved by the University of Texas Health Science Center in Houston Committee for the Protection of Human Subjects and the Memorial Hermann Hospital Research Review Committee. 

Inclusion criteria for a case were a community-acquired illness with CSF pleocytosis (leukocytes >5 cells/mm^3^) in a patient with meningitis (level 1 or 2 of diagnostic certainty for aseptic meningitis) (*15*); encephalitis (possible, probable, or confirmed) (*16*); or both. Patients with acute flaccid paralysis were included only if they had concomitant meningitis or encephalitis. Exclusion criteria were CSF positivity by Gram stain for bacteria or yeast from cytospin samples, past craniotomy, or current ventriculoperitoneal shunt.

### Data Collection and Parameter Definitions

Baseline clinical characteristics were recorded at the time the patient was seen in the emergency department and included demographic data, concurrent conditions (determined by Charlson Comorbidity Index), immunologic status, clinical features (including neurologic examination findings and Glasgow Coma Scale scores), laboratory test results, and case management. Lymphocytic pleocytosis was defined as a total CSF leukocyte composition of >50% lymphocytes. Empirical treatment was defined as initiation of antibacterial or antiviral agents before the results of the CSF cultures or molecular diagnostic methods were available. Participant outcomes were assessed at the time of discharge from the hospital by using Glasgow Outcome Scale scores (*17*): a score of 1 indicates death; 2, a vegetative state (inability to interact with the environment); 3, severe disability (unable to live independently but follows commands); 4, moderate disability (able to live independently but unable to resume some previous activities, at work or in social life); and 5, mild or no disability (able to resume normal activities with minimal to no physical or mental deficits). An adverse clinical outcome was defined as a Glasgow Outcome Scale score of 1–4.

Etiologies of meningitis and encephalitis were divided into 6 categories (bacterial, viral, fungal, mycobacterial, noninfectious, and unknown), according to the final diagnosis when participants were discharged from the hospital. For the purposes of this study, we defined peak WNV season as June 1–October 31 of each year.

### Diagnosis of WNV Infection or Other Arboviral Disease

Testing for WNV in serum and CSF was performed by enzyme immunoassay in the Memorial Hermann Hospital laboratory. We considered a positive reaction to be IgM >1.1 and IgG >1.5 units. General arbovirus serology panels (for St. Louis encephalitis, Eastern equine encephalitis, and Western equine encephalitis viruses) were performed by indirect fluorescence antibody assay at the Associated Regional and University Pathologists laboratory (Houston, TX, USA). The cutoff for a positive reaction for each virus was >1:16. A case was defined as acute WNV infection if viral genomic sequences (by reverse transcription PCR [RT-PCR]), specific IgM, or both) were detected in serum or CSF. The diagnosis was acute infection from other arboviruses when samples were positive for specific arboviral IgM in the absence of evidence of WNV infection.

### Statistical Analyses

Data were analyzed by using SPSS version 21 (IBM, Austin, TX, USA). Baseline and clinical characteristics having a clinically plausible association with suspicion of WNV and other arboviral infections were examined by bivariate analysis. We used the Fisher exact, χ^2^, and Student *t* tests. To adjust for multiple comparisons, we applied the Bonferroni correction, and we considered p<0.001 to be statistically significant. We examined continuous data by using analysis of variance.

## Results

### Cohort 

During the study period, 965 patients with a diagnosis of meningitis or encephalitis were screened for eligibility. We excluded 214 patients for the following reasons: positive Gram stain (113 patients), presence of shunt (84 patients), and postcraniotomy meningitis (17 patients). The other 751 patients were eligible: 567 were adults and 184 were children, 357 (48%) were male, and median age was 31 years (range 2 months–92 years) (Table 1). Among the 751 patients, onset of illness occurred during WNV season for 390 (52%), and 237 (32%) had encephalitis. Serum was submitted for arbovirus testing (WNV/St. Louis encephalitis virus and general arboviral panel) for 300 (40%) patients, WNV testing for 281 (37%) patients, general arboviral panel testing for 174 (23%) patients, and St. Louis encephalitis virus testing for 21 (3%) patients. A total of 725 (97%) patients were hospitalized, and adverse outcomes were seen in 85 (11%). Although the etiology was unknown for most (518 [69%]), among identifiable etiologies, the most common was viral (21%; 160 of 751 patients). Incidence of WNV infection was 4% (32 of 751 patients), which made it the third most common neuroinvasive virus causing infection during this period (following enterovirus and herpes simplex virus).

**Table 1:** Baseline characteristics and disease management, outcomes, and etiologies for 751 patients with meningitis or encephalitis, Houston, Texas, USA

Variable	No. (%)
Baseline characteristic	
Adult*	567 (75)
Male	357 (48)
White	306 (41)
Concurrent medical condition	
Charlson Comorbidity Index score >1	70 (9)
HIV infection	42 (6)
Clinical features	
Encephalitis†	237 (32)
Illness onset during West Nile virus season‡	390 (52)
Testing performed	
West Nile virus serology	281 (37)
Other arbovirus serology	174 (23)
Magnetic resonance imaging of brain	290 (39)
Management and outcomes	
Admission	725 (97)
Received empirical antibiotic treatment	582 (77)
Received empirical antiviral treatment	193 (26)
Adverse outcome§	85 (11)
Etiologies	
Unknown	518 (69)
Viral	160 (21)
Enterovirus	63 (8)
Herpes simplex virus	48 (6)
West Nile virus	32 (4)
Other¶	17 (2)
Bacterial#	43 (6)
Fungal**	15 (2)
* Mycobacterium tuberculosis*	8 (1)
Noninfectious††	7 (1)

*>18 y of age. Median age 31 (range 2 mo–92 y). †Possible, probable or confirmed diagnosis of encephalitis (*13*). ‡Jun–Oct. §Glasgow Outcome Scale score 1-4 (*14* ). ¶Varicella zoster virus (n = 7), St. Louis encephalitis virus (n = 3), acute HIV infection (n = 3), cytomegalovirus (n = 2), Epstein-Barr virus (n = 1), influenza virus (n = 1). #*Streptococcus pneumoniae* (n = 15), *Mycoplasma pneumoniae *(n = 5), *Staphylococcus aureus* (n = 3), *Streptococcus agalactiae* (n = 3), *Haemophilus influenzae* n = 3), *Escherichia coli* (n = 3), *Listeria monocytogenes* (n = 2), *Enterococcus *spp. (n = 2), *Neisseria meningitides *(n = 2), α-hemolytic *Streptococcus* (n = 1), *Streptococcus pyogenes* (n = 1), coagulase-negative *Staphylococcus* (n = 1), *Brucella *sp. (n = 1), *Treponema pallidum *(n = 1). **Cryptococcus *neoformans* (n = 14), *Histoplasma* *capsulatum* (n = 1).  ††Malignancies (n = 3), systemic lupus erythematosus (n = 1), sarcoidosis (n = 1), acute disseminated encephalomyelitis (n = 1), cerebral aneurysm (n = 1).

### Clinical Characteristics of Patients

Of the 281 patients tested for WNV, most were adult (234 [83%]; p<0.001) and white (134 [48%]; p = 0.004) (Table 2). No difference between testing status (tested or not tested) groups was found in terms of Charlson Comorbidity Index scores or HIV status. The only clinical variable significantly associated with a trend toward more WNV testing was altered mental status (76 [28%] of 281 tested for WNV vs. 84 [18%] of 470 not tested for WNV; p = 0.01). Epidemiologically, the trend was toward more WNV testing of patients with meningitis/encephalitis who were hospitalized during June–October (170/281 [60%] vs. 220/470 [47%]; p = 0.02).

**Table 2:** Baseline clinical characteristics among 751 patients with meningitis and encephalitis, by West Nile virus testing utilization, Houston, Texas, USA

Clinical characteristic	West Nile virus testing requested, no. (%), n = 281	West Nile virus testing not requested, no. (%), n = 470	p value*
Demographic			
Male	129 (46)	228 (49)	0.50
Adult†	234 (83)	332 (70)	<0.001
White	134 (48)	172 (37)	0.004
Concurrent medical conditions			
Charlson Comorbidity Index score >1	32 (11)	38 (8)	0.15
HIV infection	16 (6)	26 (6)	1.0
Clinical features			
Altered mental status	76 (28)	84 (18)	0.01
Headache	232 (83)	378 (80)	0.50
Nausea/vomiting	179 (64)	311 (66)	0.52
Seizure	22 (8)	31 (7)	0.39
Illness onset during West Nile virus season‡	170 (60)	220 (47)	0.002
Fever >38^O^C	109 (38)	214 (46)	0.08
Glasgow Coma Scale score <15	45 (16)	43 (9)	0.007
Nuchal rigidity	71 (25)	112 (24)	0.66
Rash	4 (1)	17 (4)	0.11
Focal neurologic abnormalities	63 (22)	43 (9)	<0.001
Clinical diagnosis of encephalitis§	115 (41)	122 (26)	<0.001

*p<0.001 after Bonferroni correction considered statistically significant. †>18 y of age. Median age (range) of patients tested 35 (0.2–89), not tested 29 (0.1–92); p<0.001. ‡Jun–Oct. §Possible, probable, or confirmed diagnosis of encephalitis according to the definition in (*13*).

In term of physical findings, the presence of focal neurologic deficits was associated with ordering of WNV serologic testing (63 [22%] of 281 vs. 43 [9%] of 470; p<0.001). The trend was also toward more WNV testing among patients with a clinical diagnosis of encephalitis than of meningitis (115 [41%] of 281 vs. 122 [26%] of 470; p<0.01) and among those with an abnormal Glasgow Coma Scale score (45 [16%] of 281 vs. 43 [9%] of 470; p = 0.007).

### Laboratory Results, Treatment, and Outcomes

Per inclusion criteria, all participants had undergone lumbar puncture and had evidence of CSF pleocytosis (Table 3). The trend was toward less testing among patients for whom CSF pleocytosis was higher (p = 0.017) and for those with hypoglycorrhachia (CSF glucose <45 mg/dL; p = 0.005). The ordering of serologic testing did not vary according to CSF lymphocytic pleocytosis and CSF protein level >100 mg/dL. Concomitant microbiological workups for mycobacterial, fungal, and other viral infections in CSF were performed more frequently for those in the group for whom WNV serologic testing had been ordered (p<0.001). Magnetic resonance imaging of the brain was performed for 290 (39%) of the 751 patients but was significantly more likely to be performed for patients for whom WNV serologic testing had been ordered (139 [49%] of 281 vs. 151 [32%] of 470; p<0.001).

**Table 3:** Laboratory results, treatment, and outcomes for 751 patients with meningitis and encephalitis, by West Nile virus testing, Houston, Texas, USA

Variable	West Nile virus testing requested, no. (%), n = 281	West Nile virus testing not requested, no. (%), n = 470	p value*
Cerebrospinal fluid profile			
Predominantly lymphocytes†	218 (78)	326 (71)	0.07
Protein >100 mg/dL	101 (36)	137 (29)	0.12
Glucose <45 mg/dL	27 (10)	79 (17)	0.005
Cerebrospinal fluid microbiological testing			
Bacteria, culture	272 (96)	447 (95)	0.35
Mycobacteria, culture or PCR	102 (36)	89 (19)	<0.001
Fungi, culture or antigen assay	91 (32)	71 (15)	<0.001
Herpes simplex virus, PCR	174 (62)	157 (33)	<0.001
Enterovirus, reverse transcription PCR	104 (37)	139 (30)	0.03
Magnetic resonance imaging of brain	139 (49)	151 (32)	<0.001
Management and outcomes			
Hospitalization	276 (98)	449 (96)	0.06
Empirical antibiotic treatment	215 (77)	367 (78)	0.65
Empirical antiviral treatment	98 (35)	95 (20)	<0.001
Adverse outcome‡	51 (18)	34 (7)	<0.001
Etiology			
Unknown	206 (73)	312 (66)	0.051
Viral	46 (16)	114 (24)	0.017
Bacterial	16 (6)	27 (6)	1.00
Fungal	6 (2)	9 (2)	0.80
Mycobacterial	5 (1)	3 (1)	0.43
Noninfectious	2 (1)	5 (1)	1.00

*p<0.001 after Bonferroni correction considered statistically significant. †Median leuckocytes, cells/mL (range) 134 (1–4275) for samples tested and 143 (0–49000) for samples not tested for West Nile virus; p = 0.017. ‡Glasgow Outcome Scale score 1–4 (*14*).

No significant difference was found between groups with regard to empirical initiation of antibiotic therapy; however, WNV serologic testing was associated with a higher proportion of patients who were empirically prescribed antiviral therapy and for whom outcomes were worse (p<0.001). Among all causes of meningitis and encephalitis in this study, the trend was toward less WNV testing for those for whom the confirmed etiology was viral (46 [16%] of 281 vs. 114 [24%] of 470; p = 0.017); no difference was found for other etiologic groups such as unknown, bacterial, mycobacterial, or fungal.

### WNV and Other Arbovirus Test Results

Of the 281 patients who were tested for WNV, results were positive for 32 (11%) and were compatible with acute WNV infection. All positive results for these 32 patients were obtained during June–October, as demonstrated in the epidemiologic curve (Figure). Of those with a diagnosis of acute WNV infection, equal numbers had meningitis (n = 16) and encephalitis (n = 16). General arbovirus panel testing was ordered for 174 patients, and for 11 (6%) of these, results for IgM against St. Louis encephalitis virus were positive. Among these 11 patients, positive WNV serologic results were compatible with acute WNV infection for 7, indicating the possibility of cross-reaction. However, for 4 patients, WNV serologic results were negative, compatible with their having true acute infection with St. Louis encephalitis virus.

**Figure:**
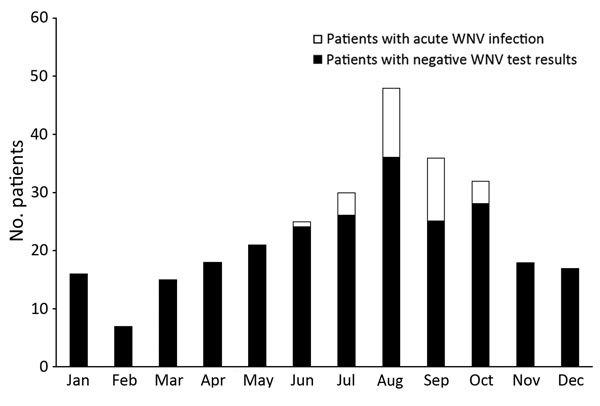
Numbers of patients for whom West Nile virus serologic testing was performed, by month, combined over 5 years (January 1, 2005, through December 31, 2010). A total of 281 patients were tested**. **

Serum was tested for WNV IgM and IgG for 168 (60%) of 281 patients; CSF was tested for IgM for 44 (16%) and serum was tested for IgM only for 40 (14%) patients (Table 4). For only 4 (1%) of the 281 patients were CSF IgM and serum IgM and IgG tested. Of the 32 patients with acute WNV infection, for 12 (38%), serum WNV IgM was positive and serum IgG was negative; and for 8 (25%), serum IgM and IgG were positive. For 4 (12%) of these 32 patients, serum and CSF IgM were both positive, and for 1 (3%), serum IgM was positive and CSF IgM was negative. RT-PCR of CSF was performed for 3 (1%) of the patients; no results were positive.

**Table 4:** West Nile virus testing and results for patients with meningitis and encephalitis, Houston, Texas, USA

Variable	Patients, no. (%)
Testing requested, n = 281	
Serum IgM and IgG	168 (60)
Only cerebrospinal fluid IgM	44 (16)
Only serum IgM	40 (14)
Serum IgM and cerebrospinal fluid IgM	17 (6)
Serum WNV IgM, IgG and cerebrospinal fluid IgM	4 (1)
Only cerebrospinal fluid, by reverse transcription PCR	3 (1)*
Only serum IgG	2 (1)
Unknown	3 (1)
Results for patients with acute West Nile virus infection, n = 32	
Serum IgM + / serum IgG –	12 (38)
Serum IgM + / serum IgG +	8 (25)
Serum IgM + / cerebrospinal fluid IgM +	4 (12)
Only cerebrospinal fluid IgM +	4 (12)
Only serum IgM +	3 (9)
Serum IgM + / cerebrospinal fluid IgM –	1 (3)

*All 3 samples had negative test results.

## Discussion

Our evaluation of the use of WNV diagnostics for patients with meningitis and encephalitis in routine clinical practice in a WNV-endemic area indicates that most cases were of unknown etiology. This finding is similar to that of the California Encephalitis Project (*18*). In our study, this finding can be explained by underutilization of testing in this patient population. The most common identifiable etiology in both studies was viral infection; however, our study detected WNV in 4% of patients and the California Encephalitis Project in only 1.2% (*18*). This difference could be explained by an epidemiologic difference (in the circulation of WNV) because more cases were reported from Texas than from California during the study periods (*1*). We found that only 37% of patients with clinically compatible illness in our study were tested for WNV, similar to 40% tested during a 2012 outbreak in Arizona, which reflects substantial underutilization of WNV testing in routine practice (*14*).

As previously reported, we found that arboviral infections were more commonly diagnosed for adults (*19*). Among children in the United States, WNV is the second most common arboviral disease, after La Crosse virus encephalitis (*19*). In our study, only 1 child received a diagnosis of WNND, but this number may be low because only 25% of children were tested for WNV. Furthermore, no patients in our study were tested for La Crosse encephalitis; such testing would have enabled a more specific comparison of accuracy. Patients with clinical features of encephalitis (altered mental status, abnormal Glasgow Coma Scale score, or focal neurologic abnormalities) were tested for WNV more frequently. However, meningitis can be found in 30%–50% of patients with WNND (*7*,*8*), similar to the 50% in our study. Therefore, meningitis should be recognized as a common manifestation of WNND, and appropriate testing should be conducted. 

All 32 cases of acute WNND occurred during June–October, similar to a US Centers for Disease Control and Prevention report that 94% of WNV cases occur during July–September (*1*). This information supports a decision to routinely send specimens collected during June–October for WNV testing. Of note, our study included all patients who had meningitis/encephalitis throughout the year; cases occurring outside WNV season might affect clinical characteristics. However, the occurrence of all WNND cases during June–October demonstrates the seasonal distribution of WNV infection and emphasizes the need to test for WNV during WNV season.

Patients with a higher level of CSF pleocytosis, hypoglycorrhachia, and lymphocytic pleocytosis >50% were less likely to get tested for WNV. Previous studies found that CSF in patients with WNND was more likely to contain <500 leukocytes/mL and to have protein and glucose levels within reference range. On the other hand, neutrophilic pleocytosis can be found in up to 40% of patients during acute infection (*7*,*20*). Thus, type of CSF pleocytosis should not influence the decision whether to submit samples for WNV testing. Patients tested for WNV infection were more likely to empirically be given antiviral therapy and to undergo evaluations for mycobacterial, fungal, and other viral infections (*21*). Moreover, patients for whom WNV serum testing was ordered were more likely to undergo brain magnetic resonance imaging and to experience adverse clinical outcomes; these factors are probably driven by a more severe clinical presentation because most patients had encephalitis. Of note, patients with a diagnosis of viral meningitis or encephalitis were less likely to be tested for WNV. This finding probably resulted from testing for WNV after receiving negative results for routine viral testing (including PCR for herpes simplex virus and enterovirus). Unfortunately, because of the design of this study, we are unable to go back and test those for whom samples were not submitted for WNV testing at the time of their illness to determine the number of cases missed because testing was not performed.

According to the Centers for Disease Control and Prevention, laboratory-confirmed acute WNV cases must meet specific diagnostic criteria; however, recent evidence of IgM persistence in WNV-positive patients has affected our ability to diagnosis true acute cases (*1*). After the initial outbreak of WNV in New York in 1999, a study found that serum WNV IgM could be detected up to 500 days after acute WNV infection in >50% of patients (*22*). A separate study in Houston also found evidence of persistent IgM; 42% and 23% of study participants were positive for IgM at 1 and 8 years after infection, respectively (*23*). As a result, patients with an isolated positive WNV IgM result in serum are considered to have a probable case. In contrast, with CSF WNV IgM testing almost all CSF IgM-positive patients converted to a negative status when CSF antibody testing was repeated within 47 days of illness onset (*24*). RT-PCR for WNV is an alternative diagnostic tool for acute infection, but its application is limited because viremia is typically undetectable by the time symptoms appear. The usefulness of RT-PCR is further complicated by the fact that median time of symptom onset to actual testing is 13 days (*25*). These findings indicate that samples to test for CSF IgM, serum IgM, and serum IgG should be sent to a laboratory for all patients suspected of having WNND, to be certain that disease onset is acute. However, our study demonstrated that samples for all 3 tests were sent for only 1% of patients. This finding reflects that, for most patients, inappropriate WNV testing was performed in clinical practice. Finally, arboviral panel diagnostics can help rule out infection with St. Louis encephalitis virus, a less common cause of neuroinvasive disease in this population.

Although supportive treatment remains the standard of care for patients with WNND, performing appropriate WNV testing may yield several benefits. An accurate diagnosis more precisely defines disease burden and epidemiology, an ongoing surveillance deficiency (*26*). Moreover, identifying WNV may lead to early detection of long-term neurologic and neurocognitive sequelae after WNND and thus enable earlier intervention (*9*).

In conclusion, WNND remains a frequent cause of acute meningitis and encephalitis in adult and child populations. The current disease burden may be underestimated because of underutilization and inaccurate choice of diagnostic tests in routine clinical practice. Samples should be submitted for appropriate WNV testing (CSF IgM, serum IgM, and serum IgG) as soon as possible for patients with meningitis or encephalitis in WNV-endemic areas during the WNV season.
